# The psychological states of people after Wuhan eased the lockdown

**DOI:** 10.1371/journal.pone.0241173

**Published:** 2020-11-12

**Authors:** Peixin Lu, Xin Li, Long Lu, Yue Zhang

**Affiliations:** 1 School of Information Management, Wuhan University, Wuhan, Hubei, China; 2 Medical Records Statistics Division of Wuhan Forth Hospital, Puai Hospital, Tongji Medical College, Huazhong University of Science and Technology, Wuhan, Hubei, China; University of Sao Paulo Medical School, BRAZIL

## Abstract

It has been two months since Wuhan eased the lockdown and the people of Wuhan have been under great pressure during COVID-19. The psychological status among healthcare workers and residents were barely know due to the lack of research after Wuhan eased of the lockdown. The purpose of this study was to assess people’s mental health and the changes after Wuhan eased the lockdown. A cross-sectional online study among citizens in Wuhan was conducted. Among 1417 participants, 387(27.0%) were frontline healthcare workers and 1035(73.0%) were residents from the general public. Their COVID-19 psychological status was evaluated using Patient Health Questionnaire-9(PHQ-9), Generalized Anxiety Disorder 7-item (GAD-7), and the PTSD Checklist-Civilian Version (PCL-C). Results show that 16.1%,22.3% and 17.2% healthcare workers and 21.2%, 16.7% and 17.2% general public had symptoms of depression, anxiety and PTSD ranging from moderate to severe. Anxiety levels were not significantly different between healthcare workers and the general public. The decreased income and the frequent social media exposure are the risk factors for general public. Compared to the early COVID-19 epidemic period, the proportion of anxiety and depression among both the general public and health workers decreased after Wuhan eased the lockdown. Our finding can be used to help the government of Wuhan to develop psychological interventions to improve the mental health of the population and work as a reference of public health guidelines for other cities with severe COVID-19 outbreak.

## Introduction

A worldwide outbreak of COVID-19 [1] which was first reported in Wuhan in December 2019. The World Health Organization announced COVID-19 as a public health emergency of international concern due to its rapid escalation [2]. As of June 14, 2020, 188 countries and regions had 7,807,734 confirmed cases of COVID-19, including more than 430,530 deaths [3].

In Wuhan, a great number of community members and healthcare workers (As HWs below) were infected with COVID-19 within a short period of time. At the same time, the Wuhan government suspended all public transportation networks and asked all residents to remain at home from January 23 to April 8 in response to the COVID-19 pandemic. This posed an unprecedented threat to the people in Wuhan, including both the HWs and general public. People were in a state of great panic due to the severe disruption to their daily life, such as shortages of daily necessities, income reduction and school closure [4], as well as the high infectivity and unclear nature of COVID-19. Within 5 months, there were 50,333 confirmed COVID-19 infections and 3,869 deaths in Wuhan. It has become the most heavily affected city in China [5].

There is a growing amount of epidemiological literature linking mental illness to exposure to huge disasters. Disasters resulting in widespread injury, life lose, income decrease and health problems usually have long run impact on psychological states [6]. Literature suggest that the unfamiliarity and uncontrollability of associated risks is related to higher susceptibility of post-traumatic stress disorder (PTSD) [7, 8]. Previous studies have reported the SARS-related PTSD symptoms in HWs and survivors in Canada [9–12], Hong Kong [13, 14], Taiwan [15, 16], and Singapore [17].

Studies in China have reported that poor mental health was commonly seen early in the COVID-19 pandemic [18–22]. But there is no study on the psychological states of Wuhan people after Wuhan eased the lockdown. It has been two months since the Wuhan eased the lockdown, and got back on track. It is time to assess the psychological states among healthcare workers and general public in Wuhan following the easing of the lockdown of the city. The purpose of this study was to explore the psychological state of people after the Wuhan lockdown eased and to assess the effectiveness of psychosocial prevention measures.

## Methodology

Our study was done remotely among HWs and general public in Wuhan from June 8 to June 18, 2020, two months since April 8 when Wuhan eased the lockdown. Prior to initiation, this study was approved by the Ethics Committee and Institutional Review Board of the Fourth Wuhan Hospital (Ref:KY2020-105-01). All participants received written consent for the first part of the questionnaire before they filling it. Our survey is anonymous and guarantees that the information will not leak out.

General public eligibility criteria included (i) lived in Wuhan from June 8 to June 18, 2020, (ii) age equal or more than 18 years. Frontline HWs are comprised of doctors and nurses from the hospitals where the COVID-19 patients were treated during the COVID-19 outbreak. We sent invitations via WeChat, QQ, and other social media networks to people who met the above criteria. If they agreed to take part in the study, we sent them a link to an online questionnaire on Wenjuanxing platform (https://www.wjx.cn/app/survey.aspx).

Previous studies have found that 50.4% and 48.3% of HWs and the general public suffer from depression in China [20, 23]. Taking these as reference, our study required at least 62 HWs and 572 residents from general public with 95% confidence interval (CI) and 5% margin of error. In total, 2238 persons participated in our survey. After excluding the participants who didn’t live in Wuhan from the 23 January to 8 April, 1417 participants were included.

### Study instruments

The questionnaire consists of five sections as follows: demographics, Patient Health Questionnaire-9(PHQ-9), Generalized Anxiety Disorder 7-item (GAD-7) and the PTSD Checklist-Civilian Version (PCL-C).

Demographic data included occupation (physician, nurse or general public), gender, age (18–25, 26–30, 31–40, or >40), geographic location (Wuhan or other), marital status (Currently married or Currently not married), educational level (undergraduate or postgraduate), income during the COVID-19 outbreak (increase, no change or decrease) and the frequency of social media exposure (few, sometimes and frequently). Income states were later grouped into “no change or increase” and “decrease” because increased income was rarely seen in the population.

We used Chinese version PHQ-9 which has been validated in Chinese population to measure the level of depression [24, 25]. A two-week recall period was used in this scale. The scale contains 9 questions measured on 4 levels: “not at all”, “several days”, “more than half days”, and “nearly every day “which we marked as “0”,”1”,”2”and “3”. The total score of the scale was interpreted as follows: normal (0–4), mild (5–9), moderate (10–14), and severe (15–27) [26]. The cutoff score for confirmed depression was 10 which have a high sensitivity(0.89) and specificity (0.97) [25]. The PHQ-9 demonstrated excellent internal consistency with α = 0.92.

The GAD-7 are widely used to scale the symptoms of anxiety severity [27]. A two-week recall was used. Seven questions were included in the GAD-7 with four options same as PHQ-9 for each one. The total score of the scale was separated into four levels, normal (0–4), mild (5–9), moderate (10–14), and severe (15–21). Higher total score indicates greater anxiety. The cutoff score for identifying the anxiety was 10 which indicated a high sensitivity(0.89) and specificity (0.82) [28, 29].

The Posttraumatic Stress Disorder Checklist (PCL) is a commonly used questionnaire to aid diagnosis of PTSD in conjunction with a clinical interview [30]. Three versions are commonly used, including PCL-M(military), PCL-C (civilian), and PCL-S (specific trauma) [31]. The PCL-C questioning symptoms associate with stressful experience and can be applied to any population [32]. The PCL-C is a 17-item self-report checklist which was responded to “1 = not at all”,”2 = a little bit”,”3 = moderately”,”4 = quite a bit” or “5 = extremely”. Higher scores represent greater self-reported PTSD. The total score of the scale was interpreted as follows: normal (17–37), mild (38–49), and (50–85) PTSD. A cutoff score of 50 used to detect the PTSD has demonstrated good sensitivity of 0.82 and specificity of 0.86 [30].

### Statistical analysis

All statistical analysis in this study was done by SPSS statistical software for Windows (SPSS 22.0). P value of 0.05 was used as the threshold of statistically significant difference. Ranking data derived from grades of depression, anxiety, and PTSD symptoms, including their total grades and percentages, were reported for each group of participants. Using Mann-Whitney U test and the Kruskal-Wallis test compare two or more than two groups. Hosmer-Lemeshow test was used in the multivariate logistic regression to get risk factors of mental health. We also reported the adjusted odds ratios (OR) and 95% confidence interval (CI).

## Results

### Demographic characteristics

Within 1,417 participants, 382(27.0%) were frontline HWs and 1035(73.0%) were from general public. Among the HWs,125(32.7%) are physicians, and 257(67.3%) are nurses. 1182(83.4%) participants are women; 838(59.2%) participants are older 30 years; 1159(81.8%) participants are married; 879[62.0%] participants don’t have an educational level higher than bachelor degree; More people had lower income during the COVID-19 outbreak. The proportion of 3 categories of social media exposure frequency exposure was 22.6% (“less”), 39.3% (“sometimes”) and 38.1% (“frequently”) respectively (Table 1).

**Table 1:** Demographic of responders.

	NO. (%)			
		Occupation		
Characteristic	Total	Physician	Nurse	General Public
**Overall**	1417(100)	125(8.8)	257(18.1)	1035(73.0)
**Gender**				
Men	235(16.6)	73(58.4)	73(28.4)	89(8.6)
Women	1182(83.4)	52(41.6)	184(71.6)	946(91.4)
**Age**				
18–25	138(9.7)	2(1.6)	10(3.9)	126(8.9)
26–30	441(31.1)	12(96)	74(28.8)	355(34.3)
31–40	552(39.0)	59(47.2)	71(27.6)	422(29.8)
>40	286(20.2)	51(41.6)	102(39.7)	132(12.8)
**Marriage status**				
Currently married	1159(81.8)	112(89.6)	197(76.7)	850(82.1)
Currently not married	258(18.2)	13(10.4)	60(23.3)	185(17.9)
**Education level**				
≤Undergraduate	879(62.0)	2(1.6)	38(14.8)	839(81.1)
≥Postgraduate	538(38.0)	123(98.4)	213(85.2)	196(18.9)
**Income**				
No change or more	247(17.4)	35(28.0)	57(22.2)	155(15.0)
Less	1170(82.6)	90(72.0)	200(77.8)	880(85.0)
**Social media exposure**				
Few	320(22.6)	19(15.2)	56(21.8)	245(23.7)
Sometime	557(39.3)	44(35.2)	90(35.0)	423(40.9)
Frequently	540(38.1)	62(49.6)	111(43.2)	367(35.5)

### Levels and extent of psychological distress on HWs

The states of mental health of HWs after COVID-19 outbreak were analyzed from two aspects. First, general anxiety and depression were evaluated using the median scores of GAD-7, PHQ-9 and PCL-C. The median (IQR) scores of the GAD-7, the PHQ-9, and the PCL-C of all HWs respondents were 5.0[1.0,9.0],6.0[1.0,9.0] and 26.0[19.0,24.0], respectively. The nurses had higher GAD-7 and PHQ-9 scores compared with physicians (e.g., IQR of GAD-7: physicians vs nurses:4.0[0–7.0] VS 6.0[2.0–9.5]). However, physicians and nurses were reported equally low scores on PCL-C scores (e.g., IQR of PCL-C: physicians vs nurses: 25.0[18.0–34.0] vs 27.0[19.0–34.0], P = 0.12) (Table 2).

**Table 2:** Score and severity categories of anxiety, depression and PTSD in healthcare workers.

		Occupation		
		No. (%)		
Score/Severity category	Total, No. (%)	Physician	Nurse	P-value
**GAD-7, anxiety**				
Score, median (IQR)	5(1,9)	4(0,7)	6(2,9.5)	0.034
Normal	168(44.0)	63(50.4)	105(40.9)	
Mild	131(34.3)	43(34.4)	88(34.2)	0.029
Moderate	54(14.1)	12(9.6)	42(16.3)	
Severe	29(7.6)	7(5.6)	22(8.6)	
**PHQ-9, depression symptoms**				
Score, median (IQR)	6(1,9)	5.0(1.0,9.0)	6.0(2.0,10.0)	0.044
Normal	161(42.1)	59(47.2)	102(39.7)	
Mild	132(34.6)	43(34.4)	89(34.6)	0.045
Moderate	51(13.4)	14(11.2)	37(14.4)	
Severe	38(9.9)	9(7.2)	29(11.3)	
**PCL-C, PTSD**				
Score, median (IQR)	26(19,24)	25(18,34)	27(19,34)	0.120
Normal	320(83.8)	108(86.4)	212(82.5)	
Mild	36(9.4)	9(7.2)	27(10.5)	0.460
PTSD	26(6.8)	8(6.4)	18(7.0)	

Second, general anxiety, depression and PTSD were evaluated using the severity of measurements. A large percentage of health care workers had anxiety symptoms (214[56.0%]) and depression (221[57.9%]), but there was low proportion of HWs with PTSD (62[16.2%]). Similar to the pattern of the median scores, compared with physicians, nurses were more likely to have symptom of anxiety and depression, but not and PTSD (Table 2).

### Psychological distress of general public

The median (IQR) scores of all general public respondents for the PHQ-9, GAD-7 and PCL-C were 5.0[1.0,8.0],4.0[1.0,9.0] and 23.0[18.0,32.0], respectively. Like HWs, a considerable proportion of general public had anxiety symptoms (529[52.1%]) and depression (473[45.7%]) and low proportion had PTSD (121[13.6%]). Compared with HWs, general public was less likely to have symptom of depression and PTSD, but not anxiety (Table 3)

**Table 3:** Score and severity categories of anxiety, depression and PTSD in general public.

		Occupation		
		No. (%)		
Score/Severity category	Total, No. (%)	Healthcare Workers	General Public	P-value
**GAD-7, anxiety**				
Score, median (IQR)	5(1,8)	5(0.75,8)	5(1,9)	0.967
Normal	664(46.9)	168(44.0)	496(47.9)	
Mild	451(31.8)	131(34.3)	320(30.9)	0.294
Moderate	208(14.7)	54(14.1)	154(14.9)	
Severe	94(6.6)	29(2.0)	65(6.3)	
**PHQ-9, depression symptoms**				
Score, median (IQR)	4(1,9)	6(1,9)	4(0,8)	<0.001
Normal	723(51.0)	161(42.1)	562(54.3)	
Mild	433(30.6)	132(34.6)	301(29.1)	< 0.001
Moderate	153(10.8)	51(13.4)	102(9.9)	
Severe	108(7.6)	38(9.9)	70(6.8)	
**PCL-C, PTSD**				
Score, median (IQR)	23(18,32)	26(19,34)	23(18,30)	<0.001
Normal	1234(87.1)	320(83.8)	914(88.3)	
Mild	110(7.8)	36(9.4)	74(7.1)	0.023
PTSD	73(5.2)	26(6.8)	47(4.5)	

### Risk factors of mental health outcomes

For HWs, after controlling confounders, multivariable logistic regression analysis showed women were more prone to anxiety(OR[95%CI]:1.48[1.02–2.16];P = 0.045)) and depression (OR[95%CI]:1.80[1.07–3.02];P = 0.026). Meanwhile, moderate social media exposure indicated lower possibility of anxiety(OR[95%CI]:0.40[0.19–0.83];P = 0.013)and depression(OR[95%CI]:0.51[0.27–0.94];P = 0.032) (Table 4).

**Table 4:** Factors influencing the mental health of HWs.

	Healthcare workers			
Category	Anxiety		Depression	
Variable	Adjusted OR (95%CI)	P value	Adjusted OR (95%CI)	P value
**Gender**				
Men	1[Reference]	NA	1[Reference]	NA
Women	1.48[1.02–2.16]	0.045	1.60[1.04–3.02]	0.034
**Age**				
18–30	1[Reference]	NA	1[Reference]	NA
>30	0.88[0.45–1.75]	0.720	0.79[0.40–1.57]	0.502
**Marriage status**				
Currently married	1[Reference]	NA	1[Reference]	NA
Currently not married	0.92[0.43–1.95]	0.820	1.41[0.69–2.89]	0.347
**Education level**				
≤Undergraduate	1[Reference]	NA	1[Reference]	NA
≥Postgraduate	0.80[0.35–1.82]	0.590	0.53[0.24–1.17]	0.114
**Income**				
No change or Increase	1[Reference]	NA	1[Reference]	NA
Decrease	1.25[0.67–2.35]	0.490	1.17[0.63–2.17]	0.622
**Social media exposure**				
Few	1[Reference]	NA	1[Reference]	NA
Sometime	0.40[0.19–0.83]	0.013	0.51[0.27–0.94]	0.032
Often	1.12[0.61–2.32]	0.611	1.71[0.87–3.36]	0.117

Multivariable logistic regression analysis of the general public showed that individuals with reduced incomes were more susceptible to anxiety (OR [95%CI]: 2.49[1.43–4.33]; P = 0.001) and depression (OR [95%CI]:1.85[1.07–3.21]; P = 0.028). Different from HWs, individuals with frequent social media exposure were more likely to suffer from anxiety (OR [95%CI]: 1.91[1.26–2.90]; P = 0.002) and depression (OR [95%CI]: 1.64[1.04–2.60]; P = 0.035) in general public (Table 5).

**Table 5:** Factors influencing the mental health of general public.

	General public			
Category	Anxiety		Depression	
Variable	Adjusted OR (95%CI)	P value	Adjusted OR (95%CI)	P value
**Gender**				
Men	1[Reference]	NA	1[Reference]	NA
Women	1.72[0.90–3.28]	0.100	1.19[0.64–2.21]	0.578
**Age**				
18–30	1[Reference]	NA	1[Reference]	NA
>30	0.93[0.67–1.29]	0.657	1.05[0.73–1.51]	0.792
**Marriage status**				
Currently married	1[Reference]	NA	1[Reference]	NA
Currently not married	0.87[0.56–1.37]	0.543	1.60[1.03–2.50]	0.037
**Education level**				
≤Undergraduate	1[Reference]	NA	1[Reference]	NA
≥Postgraduate	1.27[0.83–1.95]	0.268	1.15[0.74–1.79]	0.582
**Income**				
No change or Increase	1[Reference]	NA	1[Reference]	NA
Decrease	2.49[1.43–4.33]	0.001	1.85[1.07–3.21]	0.028
**Social media exposure**				
Few	1[Reference]	NA	1[Reference]	NA
Sometime	0.88[0.58–1.36]	0.585	1.03[0.64–1.65]	0.904
Often	1.91[1.26–2.90]	0.002	1.64[1.04–2.60]	0.035

## Discussion

This research is the first assessment of psychological states of HWs and the general public in Wuhan following the easing of lockdown in the city. This timely study evaluates the impact of COVID-19 on individual psychological states and the effectiveness of psychological interventions in Wuhan.

Our multi-center, cross-sectional survey showed that 56.0%, 57.6% and 6.2% HWs had anxiety, depression and PTSD which were lower than previous SARS studies. For example, a study that included 1275 HWs in Taiwan during the SARS outbreak showed 77.4% and 74.2% of HWs had symptoms of anxiety and depression [33]. In 2006, a study investigated the psychological states of 549 HWs located in Beijing. It was found that 10% of them had PTSD which was higher than our study [34]. Furthermore, it's worth noting that percentage of the population with depressive symptoms in this study was lower than other studies in China [20] or outside China [35]. For example, a study found that 60.0% HWs in Wuhan have depression (PHQ-9) symptoms in the early stage of COVID-19 [20].

To cope with the outbreak of COVID-19, the Government of China has carried out several prevention and control measures, such as suspension of public transportation, construction of special hospital (Huoshenshan, Leishenshan, Fangcang) gathering of medical assistance from the whole country [22]. In addition, the government in Wuhan announced it would test everyone in the city from May 15, 2020, with the goal of detecting all asymptomatic cases. With these swift measures, Wuhan’s severe COVID-19 cases dropped to zero on April 24, 2020 and no new asymptomatic cases for the first time on June 1, 2020 [36]. As the epidemiological situation improves and the workload decreases, the psychological problems of the HWs in Wuhan are alleviated.

Another possible reason of the better psychological states of the HWs could be associated with the psychological protective measures implemented in the early stage. On Jan 26, 2020, China’s National Health Commission(NHC) released the Guidelines for Emergency Psychological Crisis Interventions during the COVID-19 outbreak [37]. The Renmin Hospital of Wuhan University and Mental Health Center of Wuhan set up the psychological intervention teams in the early days of the COVID-19 pandemic, including psychosocial response team, intervention technical and medical support team, and assistance hotline teams. These four teams helped hundreds of HWs and general public to deal with mental health problem [38].

Another exciting result is that after Wuhan eased the lockdown, the general public in Wuhan had a lower proportion of moderate to severe depression and anxiety in Wuhan compared to the beginning of the COVID-19 pandemic. Mengcen et al. collected 510 and 501 residents in Wuhan and Shanghai from February 1 to 10, 2020 to investigate the psychological states of public in China. And the result showed 30.2% residents in Wuhan had moderate to severe anxiety, which is much higher than our result (21.2%) [39]. A nationwide study included 1210 general public from 194 cities in China conducted between January 31 to February 2, 2020 showed 16.5% and 28.8% of participant had moderate to severe depressive and anxiety symptoms, but in our study only 16.7% and 21.2% were identified [21]. The improvement of psychological state of people in Wuhan was obvious upon the comparison with itself during the pandemic, as well as the comparison with other cities inside and outside of China. Lzu et al. collected 8267 individuals to investigate age-related differences in measures of stress, anxiety and depression in Canada. Their results showed that in total 47.2% and 44.1% residents in Canada have moderate to severe depression and anxiety which is higher than our result [40]. Also, a Spanish study of psychological symptoms during the two-stage lockdown in response to COVID-19 showed that 26.9% and 27.5% of participants had symptoms of anxiety and depression which is also higher than our results and psychological symptoms increase as lockdown time increases [41]. The decreased rate showed in our results is partly because people's lives gradually returned to normal after Wuhan was opened again and they no longer live under worry and fear. The government has also introduced many policies to help Hubei [42, 43]. For example, it helps sell stagnant Hubei products with living streaming, helps employees resume work and production, reduces taxes and rents, and opens the night markets. These initiatives have helped the people of Wuhan recover their economy and production, and improved their confidence. According to the recently released data, the first half of the Wuhan 2020 GDP is -19.5%. Compared to the first quarter (-40.5%), the decline narrowed by 21% [43]. This result proves the effectiveness of the government's work.

Another noteworthy discovery was that anxiety levels were not significantly different between HWs and general public which reveals that the COVID-19 has severely influenced the mental health of Wuhan residents. Wuhan authorities placed the city under lockdown from Jan. 23 to April 8 to deal with the COVID-19 outbreak. During these 76 days, the daily life of people of Wuhan was severely disrupted, with isolation from families and friends, shortages of necessities, decreased wage, school closures and stay-at-home order. Previous research suggested that isolation during outbreaks may cause poor mental health because of boredom, fear, shortages of bread and butter and insufficient information [44]. Our results showed 77.8% of the general public had lower incomes than pre-pandemic. And multiple logistic regressions also showed that lower income can increase the likelihood of depression and anxiety in the general public. The other possible explanation of mental health problems is in connection with the overloaded information on social media. A previous study found that exposure to mass negative information from social media can cause PTSD [45]. A recent study found high prevalence of depression and anxiety could associate with higher frequent social media exposure [23]. Multivariable logistic regression analysis revealed that social media is a double-edged sword. Moderate social exposure decreases the incidence of mental illness of HWs, but increases it in the general public. While social media can bring timely and effective information, it can also bring you rumors and other wrong information that can pile up the anxiety and fears [4].

Agreement with earlier COVID-19 study results in the early stage COVID-19 and previous studies in SARS, our study showed that female nurses have a higher rate of psychological stress compared with doctors [20, 46], which is probably because of their heavy workload and more intensive contact with COVID-19 patients [47, 48]. The government and hospitals should take the mental health of nurses who are exposed to COVID-19 patients into account.

This study has several limitations. First, more regional, and quantitative cohort are required to understand the post-COVID-19 psychological distress to HWs and the general public. Second, the self-reported outcomes might lead to bias. However, convenience and low labor cost of the self-reported questionnaire contributed to its universality. Last, potential self-selection bias should be considered in this study. People may be reluctant to reveal their psychological problems to strangers.

## Conclusions

In conclusion, 56.0%, 47.9%, and 16.2% of the participants in Wuhan had anxiety, depression, and PTSD symptoms after easing the lockdown in Wuhan. With the pandemic in remission in China, the mental health of the HWs and general public has been improved compared with the early stage of pandemic, which proves the effectiveness of the government's work. In addition, no difference in anxiety status between HWs and the general public which is probably since the people in Wuhan have been in self-isolation for too long, with declining incomes and frequent social media network exposure.

Our study helps to the understand of the psychological states of people after Wuhan eased the lockdown. In the future, it is recommended that the government of Wuhan should develop and promote more follow-up psychological interventions, especially for the vulnerable population. It is also important to stimulate consumption, increase employment and increase people's income. Finally, our finding can also be used as a reference for public health in other cities with severe COVID-19 outbreak.

## Supporting information

S1 Questionnaires (DOCX)
