# Supplementary material for: The psychological states of people after Wuhan eased the lockdown
Source: PLoS One. 2020 Nov 12;15(11):e0241173. doi: 10.1371/journal.pone.0241173 (PMC7660514; doi:10.1371/journal.pone.0241173)
Supplement: S1 Questionnaires — (DOCX) [file pone.0241173.s001.docx]

The questionnaires were used to measure depression, anxiety and PTSD

1. Demographic data

Demographic data included occupation (physician, nurse or public), sex, age (18-25, 26-30, 31-40, or >40), geographic location (Wuhan or other), marital status (Currently married or Currently not married), educational level (undergraduate or postgraduate), income during the COVID-19 outbreak (increase, no change or decrease) and the frequency of social media exposure（few, sometimes and frequently）.

1. Depression (PHQ-9)

| **过去两周**  **Over the last two weeks** | **几乎每天**  **Nearly Every Day** | **一半以上时间**  **More than half of the time** | **有时**  **Some of the time** | **完全不会**  **At no time** |
| --- | --- | --- | --- | --- |
| 1.做什么事情都感到没有兴趣或乐趣?  Little interest or pleasure in doing things? | 3 | 2 | 1 | 0 |
| 2.感到心情低落?  Feeling down, depressed, or hopeless? | 3 | 2 | 1 | 0 |
| 3.入睡困难、很难入睡或睡太多?  Trouble falling or staying asleep, or sleeping too much? | 3 | 2 | 1 | 0 |
| 4.感到疲劳或无精打采?  Feeling tired or having little energy? | 3 | 2 | 1 | 0 |
| 5.胃口不好或吃太多?  Poor appetite or overeating? | 3 | 2 | 1 | 0 |
| 6.感觉自己很糟，或很失败，或让自己或家人失望?  Feeling bad about yourself or that you are a failure or have let yourself or your family down? | 3 | 2 | 1 | 0 |
| 7.注意很难集中，例如阅读报纸或看电视?  Trouble concentrating on things, such as reading the newspaper or watching television? | 3 | 2 | 1 | 0 |
| 8.动作或说话速度缓慢倒别人已经察觉或正好相反，烦躁或坐立不安，动来动去的情况更胜于平常?  Moving or speaking so slowly that other people could have noticed? Or so fidgety or restless that you have been moving a lot more than usual? | 3 | 2 | 1 | 0 |
| 9.有不如死掉或用某种方式伤害自己的念头?  Thoughts that you would be better off dead, or thoughts of hurting yourself in some way? | 3 | 2 | 1 | 0 |

1. General Anxiety Disorder-7 (GAD-7)

| **过去两周**  **Over the last two weeks** | **几乎每天**  **Nearly Every Day** | **一半以上时间**  **More than half of the time** | **有时**  **Some of the time** | **完全不会**  **At no time** |
| --- | --- | --- | --- | --- |
| 1.感到紧张、焦虑或烦躁  Feeling nervous, anxious, or no edge | 3 | 2 | 1 | 0 |
| 2.不能停止或控制担忧  Not being able to stop or control worrying | 3 | 2 | 1 | 0 |
| 3.对各种各样的事情担忧过多  Worrying too much about different things | 3 | 2 | 1 | 0 |
| 4.很难放松下来  Trouble relaxing | 3 | 2 | 1 | 0 |
| 5.由于不安而无法静坐  Being so restless that it’s hard to sit still | 3 | 2 | 1 | 0 |
| 6.变得容易烦扰或急躁  Becoming easily annoyed or irritable | 3 | 2 | 1 | 0 |
| 7.害怕有可怕的事情发生  Feeling afraid as if something awful might happen | 3 | 2 | 1 | 0 |

1. The PTSD Cheeklist-CivilianVersion，PCL-C

Below is a list of problems and complaints that people sometimes have in response to stressful life experiences. Please read each one carefully, pick the answer that indicates how much you have been bothered by that problem in the last month.

|  | **一点也不**  **Not at all** | **有一点**  **A little bit** | **中度的**  **Moderately** | **相当程度的**  **Quite** a bit | **极度的**  **Extremely** |
| --- | --- | --- | --- | --- | --- |
| 1. 过去的一段[压力](https://www.psychspace.com/psych/action-tag-tagname-%D1%B9%C1%A6.html)性事件的经历引起的反复发生令人不安的[记忆](https://www.psychspace.com/psych/action-tag-tagname-%BC%C7%D2%E4.html)、想法或形象?   Repeated, disturbing memories, thoughts, or images of a stressful experience from the past? | 1 | 2 | 3 | 4 | 5 |
| 1. 过去的一段压力性事件的经历引起的反复发生令人不安的梦境?   Repeated, disturbing dreams of a stressful experience from the past? | 1 | 2 | 3 | 4 | 5 |
| 1. 过去的一段压力性事件的经历仿佛突然间又发生了、又感觉到了(好像您再次体验)?   Suddenly acting or feeling as if a stressful experience were happening again (as if you were reliving it)? | 1 | 2 | 3 | 4 | 5 |
| 1. 当有些事情让您想起过去的一段压力性事件的经历时，你会非常局促不安？   Feeling very upset when something reminded you of  a stressful experience from the past? | 1 | 2 | 3 | 4 | 5 |
| 1. 当有些事情让您想起过去的一段压力性事件的经历时，有身体反应(比如心悸、呼吸困难、出汗)?   Having physical reactions (e.g., heart pounding, trouble breathing, or sweating) when something reminded you of a stressful experience from the past | 1 | 2 | 3 | 4 | 5 |
| 1. 避免想起或谈论过去的那段压力性事件经历或避免产生与之相关的感觉?   Avoid thinking about or talking about a stressful experience from the past or avoid having feelings  related to it? | 1 | 2 | 3 | 4 | 5 |
| 1. 避免那些能使您想起那段压力性事件经历的活动和局面?   Avoid activities or situations because they remind you of a stressful experience from the past? | 1 | 2 | 3 | 4 | 5 |
| 1. 记不起压力性经历的重要内容?   Trouble remembering important parts of a stressful experience from the past? | 1 | 2 | 3 | 4 | 5 |
| 1. 对您过去喜欢的活动失去兴趣?   Loss of interest in things that you used to enjoy? | 1 | 2 | 3 | 4 | 5 |
| 1. 感觉与其他人疏远或脱离?   Feeling distant or cut off from other people? | 1 | 2 | 3 | 4 | 5 |
| 1. 感觉到感情麻木或不能对与您亲近的人有爱的感觉?   Feeling emotionally numb or being unable to have loving feelings for those close to you? | 1 | 2 | 3 | 4 | 5 |
| 1. 感觉好像您的将来由于某种原因将被突然中断?   Feeling as if your future will somehow be cut short? | 1 | 2 | 3 | 4 | 5 |
| 1. 入睡困难或易醒?   Trouble falling or staying asleep? | 1 | 2 | 3 | 4 | 5 |
| 1. 易怒或怒气爆发?   Feeling irritable or having angry outbursts? | 1 | 2 | 3 | 4 | 5 |
| 1. 注意力很难集中?   Having difficulty concentrating? | 1 | 2 | 3 | 4 | 5 |
| 1. 处于过度机警或警戒状态?   Being “super alert” or watchful on guard? | 1 | 2 | 3 | 4 | 5 |
| 1. 感觉神经质或易受惊?   Feeling jumpy or easily startled? | 1 | 2 | 3 | 4 | 5 |
